# Human Prostatic Acid Phosphatase: Structure, Function and Regulation

**DOI:** 10.3390/ijms140510438

**Published:** 2013-05-21

**Authors:** Sakthivel Muniyan, Nagendra K. Chaturvedi, Jennifer G. Dwyer, Chad A. LaGrange, William G. Chaney, Ming-Fong Lin

**Affiliations:** 1 Department of Biochemistry and Molecular Biology, College of Medicine, University of Nebraska Medical Center, Omaha, NE 68198, USA; E-Mails: s.muniyan@unmc.edu (S.M.); nchaturvedi@unmc.edu (N.K.C.); wchaney@unmc.edu (W.G.C.); 2 College of Medicine, University of Nebraska Medical Center, Omaha, NE 68198, USA; E-Mail: jennifer.dwyer@unmc.edu; 3 Department of Surgery/Urology, College of Medicine, University of Nebraska Medical Center, Omaha, NE 68198, USA; E-Mail: clagrange@unmc.edu; 4 Eppley Institute for Research in Cancer and Allied Diseases, University of Nebraska Medical Center, Omaha, NE 68198, USA; 5 College of Pharmacy, Kaohsiung Medical University, Kaohsiung 807, Taiwan

**Keywords:** prostate cancer, prostatic acid phosphatase, protein tyrosine phosphatase, tumor suppressor, ErbB-2, epigenetic regulation, immunotherapy

## Abstract

Human prostatic acid phosphatase (PAcP) is a 100 kDa glycoprotein composed of two subunits. Recent advances demonstrate that cellular PAcP (cPAcP) functions as a protein tyrosine phosphatase by dephosphorylating ErbB-2/Neu/HER-2 at the phosphotyrosine residues in prostate cancer (PCa) cells, which results in reduced tumorigenicity. Further, the interaction of cPAcP and ErbB-2 regulates androgen sensitivity of PCa cells. Knockdown of cPAcP expression allows androgen-sensitive PCa cells to develop the castration-resistant phenotype, where cells proliferate under an androgen-reduced condition. Thus, cPAcP has a significant influence on PCa cell growth. Interestingly, promoter analysis suggests that PAcP expression can be regulated by NF-κB, via a novel binding sequence in an androgen-independent manner. Further understanding of PAcP function and regulation of expression will have a significant impact on understanding PCa progression and therapy.

## 1. Introduction

Human prostatic acid phosphatase (PAcP; E.C.3.1.3.2) is a prostate epithelium-specific differentiation antigen found in large amounts initially in seminal fluid. Gutman and his colleagues [1] made the critical observation that serum PAcP activity was significantly higher in prostate cancer (PCa) patients, particularly those with bone metastasis, than that in normal adult males. Subsequently in 1941, Huggins and colleagues further documented the correlation of circulating PAcP activity with prostate tumor burden [2]. Since then, serum PAcP was studied extensively as a serum marker for the diagnosis of PCa, prior to the introduction of prostate-specific antigen (PSA) [3–5]. Still, a number of studies have identified serum PAcP as a significant prognostic factor and predictor of biochemical failure and clinical recurrence of PCa [6–9]. In addition, a recent study highlighted that serum PAcP levels serve as a useful independent predictor of tumor recurrence following radical prostatectomy [10].

It has become increasingly clear that cellular PAcP (cPAcP), in addition to its role as a prostate epithelial differentiation marker [11,12], serves as an excellent measure to elucidate the molecular mechanism of cross-talk between androgens and tyrosine phosphorylation signaling involved in prostate cancer cell growth regulation [13–15]. Furthermore, cPAcP also serves as an excellent model in examining the biological activity of histidine-dependent acid phosphatases (AcPs) and their evolutionary relationship [16]. Hence, due to the biological importance of PAcP, it is imperative to understand the regulation of PAcP expression for its potential clinical applications. In light of these findings, we review PAcP biological function and compare its structure with other acid phosphatases (AcPs). We focus on the transcriptional regulation of human PAcP (hPAcP) expression to understand the molecular mechanisms that govern the expression pattern of PAcP, moving a step closer to an understanding of the altered expression pattern during PCa. Finally, we briefly describe the current understanding of PAcP as an immunogen in the immunotherapy of advanced PCa.

## 2. Biology of Human Prostatic Acid Phosphatase

### 2.1. Acid Phosphatases (AcPs)

AcPs are enzymes which hydrolyze a broad variety of small organic phosphomonoesters under acidic conditions. AcP was first described in human erythrocytes [17], and to date, at least five different AcPs have been reported in human tissues [12,18,19]. Of groundbreaking importance was work by Gutman and colleagues demonstrating the elevation of serum PAcP in advanced PCa [1].

### 2.2. PAcP Isoforms

Human PAcP is classically known as a prostate epithelium-specific 100 kDa glycoprotein [20], consisting of two subunits with molecular weight of about 50 kDa each [21]. In normal differentiated prostate epithelia, PAcP protein can be detected intracellularly as the cellular form (cPAcP) and in seminal fluid as the secretory form (sPAcP). The two forms of PAcP protein are apparently transcribed from the same gene followed by different post-transcriptional modifications. Due to the initial report on the immunological identity, it was suggested that the immunologic specificity of this enzyme resides on the protein moiety rather than the carbohydrate moiety [22,23]. These two forms of PAcP were later found to differ in some of their biochemical properties, including a partial overlapping of isoelectric points (pIs) and antigenicity [14,24–26]. Further studies have also revealed that these PAcP isoform proteins differ in glycosylation and hydrophobicity [27,28]. Antibodies with specificity against human sPAcP, but not cPAcP, have been reported [29]. Recent observations have shown the presence of a novel spliced variant form (TM-PAcP) containing a transmembrane domain in prostatic vesicles and membranes, and also in many mouse non-prostatic cells and tissues [30]. Although TM-PAcP may act as an analgesic in mice, its presence in human tissues other than prostate, and its biological function, are yet to be determined.

### 2.3. Tissue Distribution of PAcP

Immunohistochemistry (IHC) staining demonstrated that high levels of hPAcP are primarily localized in the differentiated columnar epithelial cells of prostate [31–33]. Nevertheless, positive IHC staining of PAcP, with weak reactivity, in non-prostatic tissues is also reported [32,34–41]. A study based on quantitative reverse transcription polymerase chain reaction (qRT-PCR) analyses described low PAcP mRNA expression in many non-prostatic cells including bladder, kidney, pancreas, cervix, testis, lung and ovary [42]. Recent comprehensive analyses confirmed that PAcP mRNA is overwhelmingly expressed in prostate cells when compared with the levels in other tissues analyzed [43], though its protein expression was not investigated. In cancerous cells, PAcP protein can be detected in some breast and rectal carcinomas, but at levels considerably much lower than that seen in PCa [34,44].

### 2.4. Physiological Levels of PAcP

cPAcP expression is negligible before adolescence in male prostates. In normal adults, cPAcP is found at high levels of approximately 0.5 mg/gm wet prostate tissue [12,45]. sPAcP is secreted into seminal fluid at a physiological concentration of approximately 1 mg/mL [46]. In normal healthy individuals, the plasma levels of sPAcP are on the order of 1–3 ng/mL, while its level is elevated in a disease state and correlates with the PCa stage.

### 2.5. PAcP for the Detection of PCa

Circulating levels of PAcP have long been used as a biomarker in PCa diagnosis. Though the serum PAcP level is low in healthy individuals, its level is elevated in individuals with metastatic PCa and correlates with the stage of PCa [47–49]. Hence, elevated serum PAcP level was used as an indicator for the diagnosis of PCa until the availability of gold standard PSA. In parallel, cPAcP was used to determine the prostate origin of metastatic cancers. Interestingly, the level of cPAcP corresponds inversely to PCa progression, *i.e.*, the higher the grade, the lower the cPAcP protein [50–52], despite an elevated level of sPAcP in patient circulation. Emerging observations on the inverse relationship between cPAcP level and tumor progression suggest that cPAcP can be a useful marker for predicting PCa prognosis. Results of transcriptome-based tissue microarray analyses using HG U133A GeneChip analyses reveal that cancerous specimens with Gleason scores of 6–9 have significantly decreased PAcP expression when compared to non-cancerous prostate tissue [14]. Recent studies validate that serum PAcP level, like serum PSA, is significantly increased with clinical stages of the disease [53]. Furthermore, serum PAcP is elevated in patients with bone metastases, higher than those without bone metastases, and importantly, the accuracy of circulating PAcP in detecting bone metastases is equal to PSA [53].

## 3. Structure of Human Prostatic Acid Phosphatase

### 3.1. Homology Alignments of Human PAcP Protein with Other Mammalian Species

**Figure 1:**
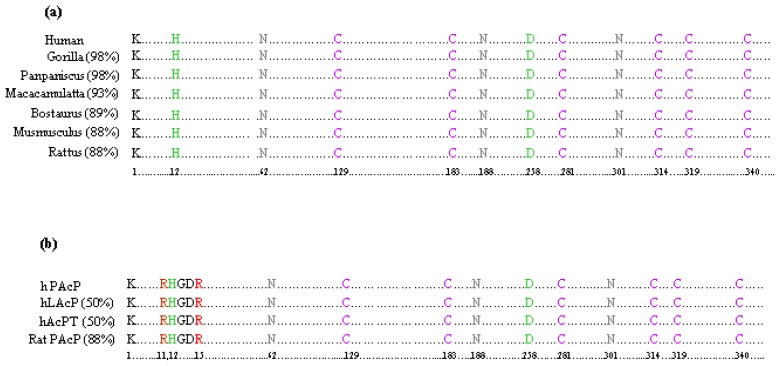
(**a**) Sequence alignment of human prostatic acid phosphatases shows that the active sites are evolutionarily conserved in closely related mammals. Only active site residues are shown with the amino acid position. Mature protein starts with lysine (K). Active site histidine (H) and aspartate (D) are highlighted in green, cysteine (C) with pink and glycosylation at asparagine (N) with gray. The number in the parenthesis indicates the percentage of similarity with hPAcP; (**b**) Sequence alignment of hPAcP with hLAcP, AcPT and rat PAcP shows that the active sites are evolutionarily conserved in closely related mammals. Only active site residues are shown with the amino acid position. Mature protein starts with lysine (K). Active site histidine (H) and aspartate (D) are highlighted in green, arginine (R) with red, cysteine (C) with pink, and glycosylation at asparagine (N) with gray. The hPAcP sequence is given in the first row, subsequent rows display the AcP sequence from other sources. The number in the parenthesis indicates the percentage of similarity with hPAcP.

Sequence analyses reveal that hPAcP closely resembles other mammalian PAcPs (Figure 1A). Human PAcP protein shows approximately 98% sequence identity with chimpanzee, pigmy chimpanzee and gorilla [54], 93% with monkey [54], 89% with cow [55], 88% with mouse [56] and rat PAcP [30]. In addition, the glycosylation sites (Asn62, Asn188 and Asn301), cysteine residues (Cys at 129, 183, 281, 315, 319 and 340) and the active site amino acid residues (His12 and Asp258) are conserved in all mammalian PAcP analyzed (Figure 1A).

### 3.2. Biochemical Properties of the Human PAcP Gene and mRNA

The gene encoding hPAcP is located on chromosome 3q21 [57]. The mRNA encodes the mature protein of 354 amino acid residues with a calculated molecular mass of 41,126 Da [58,59]. In addition to the 354 residues, the 5′-end of the coding region encodes a signal peptide of 32 amino acids. The hPAcP gene contains 10 exons. The signal peptide, and the first eight amino acids of the protein are encoded by exon 1, and the rest of the amino acids and 3′-untranslated region are encoded by exons 2 to 10 [57,60]. The hPAcP gene contains three Alu-type repetitive sequences upstream of the proximal promoter within 3kb, and two copies of the sequence in the 3′-untranslated region of the gene. In human LNCaP prostate carcinoma cells, the major transcription start site is located at 50 nucleotides upstream of the gene’s ATG codon [61]. In normal human prostate, two species of PAcP mRNA (2.4 and 3.3 kb) are found; while in prostate carcinomas including LNCaP cells, only the 3.3 kb species is detected [58,62,63]. The molecular mechanism of the lack of 2.4 kb mRNA expression remains under investigation.

In rat prostate, at least three species of mRNA (4.9 kb, 2.3 kb and 1.5 kb) are detected [64], and their differences in length are in part due to the variation in the 3′ non-coding regions [65]. The longest 4.9 kb mRNA is insensitive to androgen treatment; while the medium size 2.3 kb mRNA shows an initial increase, with a later decrease to 46% after castration [65]. Interestingly, the shortest 1.5 kb rPAcP mRNA reaches 14% after three days of castration [65]. Since the shortest mRNA transcript exhibits the highest sensitivity to androgens, it is proposed to be responsible for translating rPAcP protein [65]. Nevertheless, the possibility of the larger size mRNA species also contributing to the production of rPAcP protein cannot be ruled out. The sequence identity and the molecular significance in multi mRNA species, and also their evolutionary relationship, are unknown at present and require further investigation.

### 3.3. Structural Comparison of hPAcP with hLAcP and AcPT at mRNA and Protein Levels

Genomic analyses reveal that the human lysosomal acid phosphatase (hLAcP) gene contains 11 exons [66]. The 11th exon of the hLAcP gene encodes the COOH-terminal domain, which includes a transmembrane segment, and is found to be absent in the human PAcP gene [59]. This raises a concern regarding the origin of TM-PAcP [30]. Virkkunen *et al.* [67] demonstrated that rPAcP contains 11 exons similar to hLAcP, suggesting that rPAcP and hLAcP, but not hPAcP, may have evolved from the same ancestral gene. Nevertheless, structural analyses demonstrated that 71% of the rPAcP gene is identical to hPAcP in the 5′ region, and that exons 2 through 9 are similar in sizes [67]. Interestingly, the testicular acid phosphatase (AcPT) gene is composed of 11 exons, and the protein is predicted to have a luminal domain, a transmembrane domain and a cytoplasmic domain with the *N*-terminal end of the protein encoding a signal peptide [19].

Amino acid sequence analyses show that the hPAcP protein has at least 50% sequence similarity with hLAcP [68] and AcPT [19]. While rPAcP shows 88% sequence identity with hPAcP, the similarity between rPAcP and hLAcP is only 45% [64]. Furthermore, similar to hPAcP but unlike hLAcP or rLAcP, the rPAcP sequence lacks a membrane-anchoring domain [64]. Interestingly, alignment of the amino acid sequences of hPAcP, hLAcP, hAcPT and rPAcP indicates a high sequence similarity among these mature polypeptide chains, including the position of the cysteine residues, the *N*-glycosylation sites, and the histidine catalytic sites [68–70]. All six cysteine residues present in the overlapping areas of the mature hPAcP, rPAcP, hLAcP and rLAcP proteins are positionally conserved, suggesting that these residues are important for the tertiary structure of AcPs. The active site residues, two arginines (Arg11 and Arg15), one histidine (His12) and one aspartate (D258) in hPAcP, are also conserved in these AcPs (Figure 1B). Furthermore, the antigenic determinants for both hPAcP and hLAcP are similarly located at both of the terminal regions with a higher similarity on the NH_2_-terminal peptide than the COOH-terminal site [71]. Nevertheless, it should be noted that these two proteins exhibit a low immune cross-reactivity. The unique immunological activity of hPAcP protein allows it serving as a potent antigen in immune therapy of advanced castration-resistant PCa.

### 3.4. Structural Analysis of the hPAcP Protein

The precursor form of hPAcP protein is composed of 386 amino acids. After the cleavage of the 32 amino acid signal peptide, the mature PAcP protein (354 amino acids; Mr 41,126 Da) becomes catalytically active. Native hPAcP protein is a dimer [20,21,72], consisting of two subunits [73]. Sequence analyses have revealed that each monomer contains three asparagine-linked glycosylation sites (62, 188 and 301), and six cysteine residues forming two disulfide bonds (Cys129–340 and Cys314–319) and two free residues (Cys183 and Cys281) [74–76]. The glycosylation and disulfide linkages support the stability of hPAcP protein.

Secondary structural analyses demonstrated that hPAcP is composed of 44% α-helix (16 helices; 158 residues) [77], 12% β-strand (ten strands; 45 residues) and the rest are loops and β-turns [78]. Denaturation-renaturation and subunit reassociation studies showed that hPAcP activity may depend on dimer formation [73]. The three dimensional crystal structure of hPAcP protein revealed that each subunit has two domains: The larger domain is α/β type composed of a central seven-stranded mixed β-sheet with helices on both sides; while, the second, smaller domain contains six α-helices and is formed mostly by long-chain excursions from the first domain and α-loop with no secondary structural elements [76]. The PAcP active site residues His12 and Asp258 are found to be located between the domains. Site-directed mutagenesis studies have shown that His12 and Arg11 are essential for catalysis, while the substitution of residues corresponding to Arg15, Arg79, His257 and Asp258 severely impairs the catalytic activity [75]. Furthermore, His12 acts as an acceptor of the phosphate group, Asp258 is a proton donor for the substrate-leaving group, and His257 may participate in substrate binding, or may facilitate the breakdown of the phosphoenzyme complex [75]. It was also observed that the presence of His12 in the conserved “RHGXRXP” motif [74] revealed the enzymatic dephosphorylation property of PAcP through the formation of a phosphohistidine intermediate [16,74,79,80].

### 3.5. Biochemical Characterization of hPAcP Isoforms and Allosteric Regulation

Several PAcP isozymes with different molecular weights or pIs have been reported [24,25,28,81–84]. Vihko [24] proposed that a minor species of purified PAcP protein from prostate tissue is the authentic cPAcP in prostate cells and it exhibits only partial cross-reactivity with Ab to sPAcP. Lin *et al.* [82] purified an acidic form of PAcP protein from PCa tissue and identified it as the cancer-associated form of the enzyme. This acidic form of PAcP is highly glycosylated, including sialylation, which contributes to the elevation of plasma PAcP in PCa patients.

Analyses of PAcP protein isolated from human seminal fluid reveal two isoforms of the enzyme, PAcP-A and PAcP-B [71]. PAcP-A is the major isoenzyme and PAcP-B represents the minor species. Each isoform has multiple pI values of 5.05–5.35 *vs.* 5.05–5.12, substrate and inhibitor specificities, respectively. Both isoenzymes consist of two 50 kDa subunits. PAcP-B is found to have three components, designated as α, β and γ, with a molar ratio of 2:1:1. Interestingly, PAcP-A contains only α components. Thus, PAcP-A is a homodimer, containing two identical α subunits with high specific activity, whereas PAcP-B is a heterodimer (αβ or αγ) with low activity [71]. Lee *et al.* [71] suggested that the α-subunit functions as the catalytic subunit of PAcP and the functions of the β- and γ-subunits are still not known. It is biochemically significant to determine if the β- and/or γ-subunit are regulatory subunits. In parallel, van Etten *et al.* [74] showed that purified PAcP protein from seminal fluid exhibits different cleavage forms at the *C*-terminal sequence. This finding raises the possibility that β- and γ-subunits are partially cleaved products of α-subunit.

Importantly, enzymatic characterization by serial dilutions of purified PAcP protein reveals that the monomeric PAcP protein exhibits very low phosphatase activity and the dimerization of the mature PAcP protein allows PAcP to obtain the full catalytic activity [85]. This dimerization of the PAcP monomer exhibits the allosteric activation phenomenon [85]. This activation by dimerization is similar to the requirement of oligomerization for receptor protein tyrosine kinase (RPTK) activation. Thus, oligomerization plays an important role in the activation of PAcP, a histidine-dependent tyrosine phosphatase [16]. Results from Porvari *et al.* [86] indicate that Trp106 and His112 residues of mature rPAcP are involved in regulating its dimerization and subsequent activation. Thus, hPAcP may serve as an interesting model in studying the molecular mechanism of PAcP dimerization relating to its biological function.

## 4. Biological Function of Prostatic Acid Phosphatase

### 4.1. Cellular PAcP as a Tumor Suppressor

**Figure 2:**
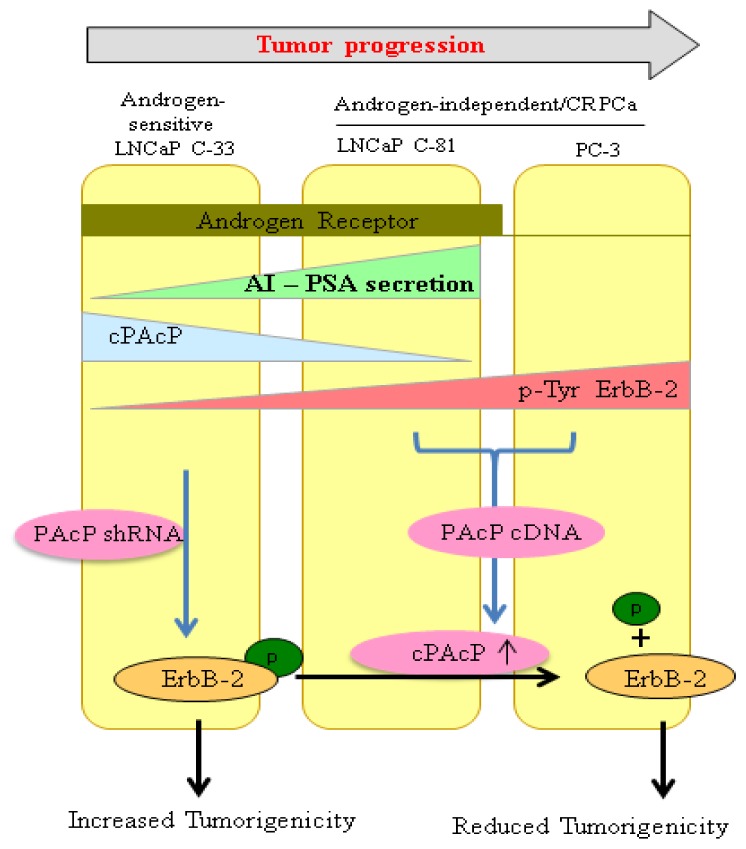
Association of cPAcP and ErbB-2 phosphorylation levels in androgen-sensitive (AS) LNCaP C-33 and androgen-independent (AI) LNCaP C-81 and PC-3 cells. Knockdown of endogenous PAcP in LNCaP C-33 cells leads to increase ErbB-2 tyrosine phosphorylation and tumorigenicity. Conversely, ectopic expression of cPAcP expression in AR-positive LNCaP C-81 and in PAcP-null PC-3 cells restores their androgen sensitivity, decrease the growth rate and tumorigenicity.

PAcP has a high level of expression in well-differentiated normal human prostate epithelial cells, which is in accordance with a slow growth rate [12,45]. Despite the elevated level of sPAcP in PCa patient sera, several studies clearly show that the cPAcP level is decreased in PCa archival specimens, compared with the adjacent non-cancerous cells [14,87–90]. Hence, it is proposed that prostate epithelia having a low level of cPAcP expression are at a high risk of carcinogenesis [87]. The notion is further supported by the observation that in human PCa cell lines, the cellular level of PAcP is inversely correlated with the proliferation rate [91]. In LNCaP and MDA PCa cell lines, upon passage, decreased cPAcP expression correlates with increased growth rates of LNCaP C-81 and MDA PCa2b AI cells [14,92–94]. Conversely, the expression of cPAcP by cDNA transfection into LNCaP C-81 and PC-3 cells diminishes their growth rates [15,89,95]. Furthermore, decreased endogenous PAcP expression by antisense cDNA or siRNA in LNCaP C-33 cells is associated with increased growth rates and tumorigenicity [14,15] (Figure 2). Further, a single intratumoral injection with the expression vector encoding wild type PAcP protein, but not the inactive mutant, suppresses the xenograft tumor development by androgen-independent LNCaP C-81 cells [96]. Supportively, in 1α,25-dihydroxyvitamin D3 (the active form of Vitamin D) treated androgen-independent (AI) LNCaP C-81 cells, cellular PAcP level was increased, which in-turn decreases PCa cell proliferation by selectively reducing tyrosine phosphorylation [97]. In parallel, stable PAcP cDNA-transfected subclonal cells had reduced tumor development when compared with control LNCaP C-81 cells [96]. These results collectively demonstrate that the active form of cPAcP has a significant tumor suppression effect, not only in *in vitro* cell cultures, but also in the mouse xenograft tumor model.

### 4.2. sPAcP: Functions beyond Tumor Suppressor

While sPAcP in seminal fluid is proposed to be involved in fertility, in part by affecting the motility of sperm [98], this role of sPAcP has been questioned [99,100]. Additionally, a sPAcP fragment forming the amyloid fibrils called semen-derived enhancer of viral infection (SEVI) may enhance HIV transmission [101,102]. The biological activity of sPAcP requires further investigation.

## 5. Regulation of PAcP Expression

### 5.1. Effects of Multi Factors on PAcP Expression

Cell density has a significant effect on the expression of functional genes involved in cell growth regulation [103–105]. Pioneer studies revealed that human cPAcP protein level is correlated with the differentiation of the human prostate gland, which is associated with increased cell density and confluence [11,12]. In LNCaP human prostate carcinoma cells and canine prostate primary epithelia, cPAcP protein level is elevated with cell density increases [106–108] and PAcP mRNA levels are decreased in LNCaP cells [95,108]. It was thus hypothesized that in high density-cultured cells, the accumulated level of PAcP protein suppresses the transcription of PAcP gene by a feed-back mechanism, or decreases the half-life of its mRNA [109].

The expression of PAcP has been thought to be directly regulated by androgens [110]. Recent studies showed that the PAcP gene promoter does not contain a functional androgen-responsive element, differing from PSA [110–112]. Further studies revealed that androgens can up-and downregulate PAcP mRNA, depending on cell densities [109]. Additionally, growth factors such as EGF and TGF-α show negative regulatory effects on PAcP mRNA. On the other hand, TGF-β_1_, which inhibits normal prostatic epithelial cell growth [113,114], upregulates the expression of PAcP mRNA [115]. All these studies together support the notion that PAcP expression is regulated in an androgen-independent, manner [110–112]. Further studies are needed to elucidate the molecular mechanism of regulating PAcP expression.

Secretion of PAcP protein was observed when LNCaP cells were cultured in media devoid of steroids and growth factors [92,110,116,117]. Additionally, sPAcP secretion is mediated by a regulatory process including androgens, Rab27a, PI3K and PKC [118–121]. However, the elevated sPAcP protein level can be explained by the increased half-life period of sPAcP in slow-growing, high density cells. The molecular mechanisms responsible for the lower PAcP mRNA level in high density cells is not yet clear.

### 5.2. Transcriptional Regulation of hPAcP Gene Expression

Transcriptional regulation of gene expression is primarily achieved by modulation of its promoter activity. The formation of a transcription complex depends upon the specific association of multi-transcription factors which can lie either within close proximity of the promoter or at far distance. The expression of PAcP is regulated by the coordination of the cis-regulatory elements of its promoter [61,67,112,122] and transcription factors [123], and also epigenetic regulation [124].

Sequence analyses reveal that the human PAcP gene promoter DNA, within 3 kb upstream of the coding region, lacks the canonical TATA box and the GC box, and that there are five putative androgen response elements (AREs) in this gene [61,67]. While the PSA promoter is upregulated by androgens [125–127], PAcP expression is not androgen-dependent and the AREs are not functional [113,114,122]. Utilizing two androgen receptor (AR)-negative, androgen-independent PCa cell lines, PC-3 and DU 145, the reporter gene assay showed that the PAcP promoter is highly active in those cells in the absence of AR cDNA co-transfection or the addition of androgens. These results clearly demonstrate that the promoter activity of the PAcP gene is regulated by an androgen-independent manner [110,111,122].

It has been demonstrated that the 1.4 kb promoter DNA sequence (from −1356 to +87) exerts an inverse correlation with the growth of LNCaP cells [63]. Using human PCa cell lines PC-3 and DU 145, PAcP-null cells, a region upstream of the PAcP gene from −2899 to +87 bp was linked to the reporter of the chloramphenicol acetyltransferase (CAT) gene. Analyses by sequential deletions of the sequence reveal that the region between −1258 and −779 contains a positive regulatory element(s) by enhancing the PAcP promoter activity in PC-3 and DU 145 human PCa cells, but not in non-prostate cells, such as WI-38 lung diploid cells, A-431 epidermoid carcinoma cells and HeLa cervix epitheloid carcinoma cells [112,122]. Further studies indicate that PAcP transcriptional activation requires at least 200 bp of the 5′-flanking sequence [112]. The sequence further upstream, such as from 5′ to −799 bp, does not show any significant effect on the transcriptional activity. It is still possible that this region of −779 to +87 contains some unknown sequences which are involved in the regulation of PAcP promoter activity, and the actual enhancer region might extend towards the transcription start site.

Deletion analyses of the PAcP promoter indicate that the −1305/+87 bp proximal sequence exhibits the highest reporter gene activity in both human PCa cell lines LNCaP and PC-3. This activity is suppressed by two regions, including −2583 to −1305 and −2899 to −2583 fragments, indicating that these two fragments contain negative cis-regulatory elements. Furthermore, there is a cooperative effect between these two regions [112]. Interestingly, the second suppressor (−2899 to −2583) is more active in PC-3 cells than in LNCaP cells, and has a position-independent activity. This fragment also exhibits orientation-independent inhibitory activity in both PC-3 and HeLa cells. Therefore, the high level of the cell-specific expression of the PAcP gene is apparently governed by the positive element, but not by the negative element [122].

**Figure 3:**
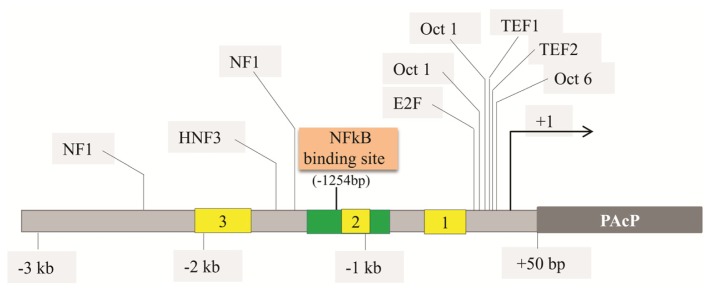
The schematic representation of the PAcP gene promoter. The transcription starts at +1 and the grey box indicates the translational region for PAcP protein (starts at +50 bp). The yellow boxes indicate the Alu repeat. The novel NF-κB binding site is identified in the positive regulatory domain (Green box; at −1245 bp upstream). The computer analysis of the sequence shows at least nine additional putative transcriptional binding sites.

Sequence analyses on the PAcP positive regulatory fragment from −1356 to −779 show that there is no consensus binding site for ubiquitous transcription factors, except for AP1 and CREB proteins. However, the putative AP1 binding sequence (PSD sequence) in the cis-active region of the PAcP promoter does not interact with the AP1 protein, nor can its consensus oligonucleotides compete with the PSD oligonucleotide in the DNA-protein complex formation [123]. Additionally, the putative CREB-binding site is not within the protein interaction domain in footprinting assays [123]. The 577 bp fragment (−1356 to −779) contains a non-consensus nuclear factor κB (NF-κB)-binding site, which is required for NF-κB up-regulation of PAcP promoter activity in PCa cells. In addition, in PC-3 cells, the TNF-α could stimulate the transcriptional activity of p1356 about 20-fold higher than p779. However, TNF-α fails to have the same effect in HeLa cells [123]. Different NF-κB dimers, homo- or hetero-dimeric complexes of various subunits, can bind to the known κB sites bearing the consensus sequence GGGRNNYYCC or GGRRNNYCCC [128]. Nevertheless, gel shift experiments and mutation analyses reveal that AGGTGT (−1254 to −1249), in the promoter of human PAcP gene, is the core sequence for NF-κB-binding and activation. It is a novel binding sequence for NF-κB located inside the cis-active enhancer element of the PAcP promoter [123] (Figure 3). Interestingly, this sequence also appears in several genes with high levels of expression in normal prostate epithelial cells including PSA, Nkx-3.1, and MIC-1 [123] and its biological significance requires further investigation.

### 5.3. Epigenetic Regulation of PAcP

Like other solid tumors, PCa is also driven by epigenetic changes such as DNA methylation and histone modifications in tumor suppressor genes. Better understanding of epigenetic changes of tumor suppressor genes and the treatment-induced restoration of tumor suppression gene function have made them attractive targets for prostate cancer treatment [129,130].

**Figure 4:**
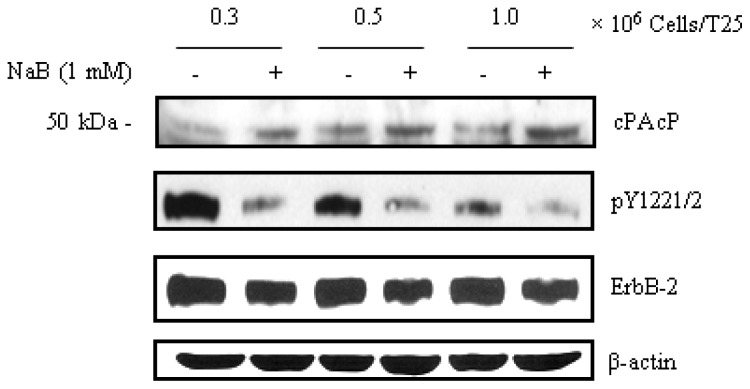
Effects of NaB on cPAcP protein expression and ErbB-2 tyrosyl phosphorylation. LNCaP C-81 cells were plated in different cell densities (0.3, 0.5 and 1 × 10^6^ cells/T25) in regular culture conditions for 2 days and then treated with 1 mM NaB for 48 h. The cells were harvested and the total protein was subjected to western blot analyses. NaB effects on cPAcP protein expression and ErbB-2 phosphorylation at Tyr1221/2 levels were shown. β-actin was detected as a loading control. The data shown is a representative from three sets of independent experiments.

Several lines of evidence demonstrated that cPAcP functions as a negative growth regulator of prostate epithelia [82,90,96,131], *i.e.*, cPAcP protein inversely correlates with the growth rate of PCa cell lines [2,5,45,90,96]. Western blot analyses in LNCaP C-81 human PCa cells shows the restoration of cPAcP upon HDAC inhibitor treatments including sodium butyrate (Figure 4), trichostatin A (TSA), and valproic acid (VPA) [124]. Further, in NaB- and VPA-treated cells, increased cPAcP protein concurs with decreased ErbB-2 Tyr1221/2 and Tyr1248 phosphorylation (Figure 4) [124]. This dephosphorylation function of cPAcP is at least in part due to its intrinsic protein tyrosine phosphatase activity [95,131,132]. In addition, the acetylation of histones H3 and H4 were greatly upregulated by VPA treatment in LNCaP C-81 cells [124]. Interestingly, HDAC inhibitor-treated PCa cells also increase their androgen responsiveness of growth stimulation [124]. Collectively, these data indicate that cPAcP is involved in HDAC inhibitor-induced growth suppression and functions as a tumor suppressor gene in regulating PCa progression and metastasis [124]. Further understanding of the restoration of this tumor-suppressor protein, cPAcP, will lead to a new avenue for treating patients with advanced CR PCa.

## 6. ErbB-2/HER-2/neu (ErbB-2) Signaling and Androgen Sensitivity Regulated by cPAcP

ErbB-2 protein, one of the most studied type-1 receptor tyrosine kinases in human cancers, has been found to be elevated in a small subpopulation of advanced PCa patients under androgen deprivation therapy (ADT). Although there is not a known ligand to activate ErbB-2, autophosphorylation of ErbB-2 at different tyrosine residues has been shown to transmit diverse biological responses.

Several lines of evidence, including studies on xenograft animal models, support the notion that elevated ErbB-2 specific activity plays a critical role in CR PCa progression [133–136]. In parallel, it is shown that the overall tyrosyl phosphorylation level of ErbB-2 protein is inversely correlated with cPAcP activity [13,15,92,131]. Ectopic expression of cPAcP by cDNA transfection restores androgen sensitivity of AR-positive, AI PCa cells. Conversely, knockdown of cPAcP expression by siRNA in AS PCa cells leads to increased cell growth in steroid-reduced conditions with a concurrent increase in tyrosine phosphorylation of ErbB-2 [14,15]. Importantly, the cPAcP-knockdown cells develop xenograft tumors in female mice in which the circulating testosterone level is similar to that in castrated male mice [15]. In PAcP knock-out mice, the prostate develops carcinomas spontaneously and the protein tyrosine phosphorylation activity is increased [137,138]. The increased p-Tyr level of ErbB-2, at least in part due to decreased cPAcP activity, is associated with decreased androgen responsiveness of PCa cells [89,92,134]. This is similar to the observation in advanced CR prostate carcinomas in which cPAcP mRNA and protein levels are diminished [89,139,140]. Thus, cPAcP is involved in regulating the androgen sensitivity of PCa cells.

cPAcP can dephosphorylate human ErbB-2 at different sites. In AI human LNCaP C-81 and MDA PCa2b AI PCa cells, the phosphorylation levels of Tyr1221/2 and Tyr1248 are elevated [15]. Conversely, the ectopic expression of WT cPAcP in LNCaP C-81 cells by cDNA transfection decreases the phosphorylation levels of Tyr1221/2 and Tyr1248 in a dose-dependent manner and the cells restore the androgen sensitivity [15,89,134]. Apparently, Tyr1221/2 and/or Tyr1248 of ErbB-2 can be regulated by cPAcP and are involved in regulating DHT sensitivity [13]. Due to the clinical importance of androgen sensitivity in PCa, further studies are needed to determine the specific site in this mode of regulation.

**Figure 5:**
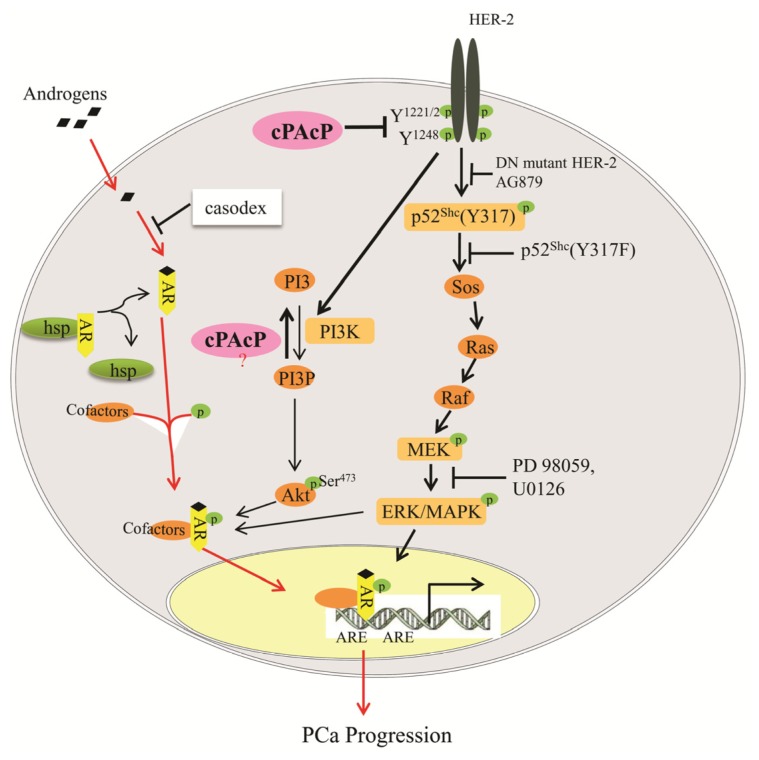
Schematic representation of ErbB-2 signaling and androgen sensitivity regulated by cPAcP in prostate cancer cells. The solid red arrow indicates the classical ligand dependent activation of androgen receptor (AR) pathway. Unbound AR resides in the cytosol in association with heat shock proteins (hsps). Androgen (DHT) enters into the cytoplasm and binds to the AR by displacing associated hsps, which allows the AR to enter into the nucleus, where it dimerizes, recruits various co-regulatory proteins and binds to the androgen response element (ARE), leading to the transcriptional regulation of the target gene. Solid black arrows indicate one of the major cPAcP-regulated pathways in prostate cancer cells with clinical significance. Progression of androgen-sensitive PCa cells towards androgen independence is accompanied by an early decrease/loss of cPAcP expression in PCa cells, results in hyperphosphorylation of HER-2 at tyrosine residues (1221/2 and/or 1248), leading to androgen-independent cell proliferation. Activated HER-2 can transduce its signals via p52Shc (blocked by dominant-negative (DN) HER-2 cDNA transfection or an HER-2 inhibitor, AG879) to activate the downstream ERK/MAPK pathway (blocked by p52Shc Y317F mutant cDNA transfection or an MEK inhibitor, PD 98059 and U0126) through Ras/Raf mediation. These events could also lead to AR phosphorylation and activation, resulting in an increase in androgen-independent cell proliferation. Activated HER-2, via Akt, may also phosphorylate AR. Alternatively, the loss of cPAcP expression results in the accumulation of PI3P, which may lead to activation of the Akt pathway.

Overexpression of ErbB-2 also enhances AR activity by activating ERK/MAPK, a non-steroid-dependent AR activation pathway [133,141]. This AR activation leads to the emergence of AI PCa cells that can survive and proliferate in an androgen-ablated environment, leading to the recurrence of PCa [142,143]. Supportively, ERK1/2 are activated in advanced prostate carcinomas and AI PCa cells in which cPAcP is decreased or null, suggesting that decreased cPAcP results in activated ErbB-2 and down-stream ERK1/2 signaling for CR PCa progression [135,144,145]. In parallel, in the face of low or null PAcP, both ErbB-2 and Akt are activated, resulting in increased cell proliferation [15]. Subsequently, Akt can phosphorylate AR at Ser213 and Ser791 sites and abrogation of Akt signaling also abolishes the AI survival and growth of these cells [146]. Thus, it is possible that cPAcP dephosphorylates PI3K and/or PI3P and thus blocks Akt activation. Additionally, phosphorylation of p52Shc at Tyr317 mediates androgen-stimulated PCa proliferation [147], the ligand-activated AR interacts with active STAT5 and enhances its nuclear translocation, and STAT5 can, in turn, increase the nuclear translocation of AR in these PCa cells [148]. Conversely, in AI PAcP-null PCa cells, ectopic expression of PAcP results in decreased pTyr1221/2 of ErbB-2 and blocks its downstream signaling, which leads to cell growth suppression through the inactivation of p52Shc, ERK1/2, Akt, Src, STAT-3, and STAT-5 [15]. Together, these results provide an explanation for the clinical phenomenon that in PCa cells, the decrease of cPAcP expression in advanced PCa cells contributes to the activation of ErbB-2, primarily by phosphorylation regulation. This leads to ERK/MAPK, Akt, STAT-3 and STAT-5 activation and advanced PCa cell survival, proliferation and PSA production under androgen-ablated conditions (Figure 5). Thus, the interaction between cPAcP and ErbB-2 regulates the downstream signaling by ErbB-2 and is involved in controlling the basal as well as the androgen-stimulated proliferation of human PCa cells [15,134].

## 7. PAcP as a Therapeutic Agent

With the limited efficacy of conventional radiotherapy and chemotherapy and with significant morbidities of surgical procedures in advanced prostate cancer, other approaches for treating clinical PCa are under active consideration. Recent research supports the notion that immunotherapy is a potential therapeutic strategy for prostate cancer as this epithelial malignancy has special features, including: The slow growth rate, the ability to induce auto antibodies, the expression of tissue-specific antigens, and susceptibility to antitumor immune response [149–154].

Studies with experimental animals have indicated that cPAcP has potential for therapeutic effect against PCa. A single intratumoral injection of a vector encoding the wild type PAcP protein, but not an inactive mutant, results in suppression of the growth and progression of xenograft prostate tumors [96]. Similarly, the injection of DNA vaccine encoding PAcP protein elicits antigen-specific CD8 T cells in rodents [155,156]. Thus, the restoration of cPAcP expression in PCa cells may provide a new avenue for treating CR PCa in which the expression of cPAcP is decreased.

Using a patient’s own immune cells, immunotherapeutic vaccines induce an antitumor response [157] by targeting tumor-associated antigens (TAAs) or by disrupting molecular pathways that promote tumor growth [158,159]. Despite the low efficacy with PSA as the immunogen tested initially, PAcP exhibits unique immune reactivity, with tissue-specific expression, and thus serves as a useful antigen in developing immune therapy towards PCa. Supportively, the naturally occurring PAcP-specific T-helper cells are found in about 11% of patients with PCa [160]. In parallel, the dendritic cells loaded with an engineered antigen-cytokine fusion protein consisting of PAcP and GM-CSF are capable of inducing a potent cellular immune response, *in vivo*, to rodent tissues and tumors that express PAcP [161].

Based on the above described preclinical observations, a dendritic cell product consisting of autologous dendritic cells loaded with the human PAcP-GM-CSF fusion protein has been developed [161,162]. Sipuleucel-T is an autologous active cellular immunotherapy product composed of autologous peripheral blood mononuclear cells (PBMCs), including antigen-presenting cells (APCs) with a recombinant fusion protein PA2024 (full length PAcP) linked to an adjuvant (granulocyte macrophage colony-stimulating factor). The autologous immunotherapeutic product infused to the patients is thought to activate PAcP specific CD4+ and CD8+ T cells, which mediate the antitumor response in prostate cancer patients [163].

An earlier multiple phase I/II trial of Sipuleucel-T in thirty one metastatic and non-metastatic PCa patients demonstrated a 100% T-cell proliferation response to the antigen PA2024 and 38% to native PAcP with PSA declines of ≥25% in six patients. The median time to disease progression was about 29 weeks [161]. In an earlier phase II study, Sipuleucel-T treatment resulted in significant development of antigen-specific cellular levels in all patients from week 4 throughout the follow-up period. PSA declines of about 25% in two patients, and a negligible amount in one patient, were observed, with median time to disease progression of about 4 months [164]. The later phase II study also shows survival benefit with Sipuleucel-T [165,166]. The larger multi-institutional phase III (IMPACT or D9902B) study with 512 metastatic CR PCa revealed 4.1 months of additional survival benefit for Sipuleucel-T treated patients when compared with placebo treated patients [162]. Further, the meta-analysis from all three phase III trials (D9901, D9902A and IMPACT) showed Sipuleucel-T treated groups had higher number of pain-free patients [167], with T-cell activation and enhanced cytokine production observed in PA2024 cultures but not in GM-CSF cultures [168]. Importantly, another meta-analysis revealed that the median survival difference for African Americans was 30.7 months when compared to 4.1 months in the overall cohort, suggesting that African American patients may benefit more from Sipuleucel-T [169]. As the sample size is small (5.8%) from the total population [169], further investigation is needed. Interestingly, a study on comparative analysis of the toxicity and survival benefit revealed Sipuleucel-T has relatively low toxicity and higher median survival benefit when compared with other FDA approved PCa treatment agents [170]. In addition, a recent study suggests that Sipuleucel-T treatment shows the most benefit for the patients with less advanced diseases [171].

## 8. Conclusions

Circulating PAcP activity has a long history of serving as a surrogate marker for diagnosing PCa as well as for evaluating the efficacy of ADT for advanced PCa. Several lines of evidence collectively support the importance of cPAcP enzyme in PCa, particularly during castration-resistant progression, and its role in regulating the growth of prostate epithelial cells through its neutral PTP activity by dephosphorylating p-Tyr of ErbB-2. The theoretical and experimental approaches based on peptide studies confirm that PAP could dephosphorylate ErbB-2 protein. The structural analyses and mutant experiments further identify the active site residues, His12 and Asp258, responsible for the dephosphorylation of ErbB-2 protein. In addition, the generation of PAcP knockout cell lines and PAcP “knock-out mouse” models have supported the notion that disrupting the expression of PAcP leads to tumor development in prostate tissue, which suggests cPAcP signaling as a potential therapeutic target for advanced PCa. Therefore, due to the potential importance of the PAcP gene as a novel tumor suppressor in prostate cancer, and the promising clinical trial results from the cPAcP immunotherapy, further investigation on the biology of cPAcP expression may provide novel valuable insights into its inhibitory role in PCa for potential therapeutic applications.

## Abbreviations

AcP: acid phosphatase
AcPT: testicular acid phosphatase
AI: Androgen-independent
AS: Androgen-sensitive
cPAcP: cellular prostatic acid phosphatase
CR PCa: castration-resistant prostate cancer
DHT: 5α-dihydrotestosterone
EGF: epidermal growth factor
EGFR: EGF receptor
HER-2/ErbB-2/neu: human epidermal growth factor receptor-2
FBS: fetal bovine serum
HDAC: Histone deacetylase
IHC: Immunohistochemistry
hLAcP: human lysosomal acid phosphatase
hPAcP: human prostatic acid phosphatase
PCa: prostate cancer
pI: isoelectric point
PI3K: phosphoinositide 3-kinase
PKC: protein kinase C
PSA: prostate-specific antigen
PTP: protein tyrosine phosphatase
p-Tyr: phosphotyrosine
qRT-PCR: quantitative reverse transcription-polymerase chain reaction
sPAcP: secretory PAcP
TM-PAcP: transmembrane PAcP
Tyr-P: tyrosine phosphorylation.
